# Clinical characteristics of patients with bronchiectasis with nontuberculous mycobacterial disease in Mainland China: a single center cross-sectional study

**DOI:** 10.1186/s12879-021-06917-8

**Published:** 2021-12-06

**Authors:** Hongjun Yin, Xiaoying Gu, Yimin Wang, Guohui Fan, Binghuai Lu, Min Liu, Chunlei Wang, Bin Cao, Chen Wang

**Affiliations:** 1 Xuanwu Hospital of Capital Medical University, Beijing, China; 2 Department of Pulmonary and Critical Care Medicine, Center of Respiratory Medicine, National Clinical Research Center of Respiratory Diseases, China-Japan Friendship Hospital, East Yinghua Road, Chaoyang District, Beijing, China; 3 Department of Infectious Diseases, Beijing Luhe Hospital, Capital Medical University, Beijing, China; 4 Department of Institute of Clinical Medical Sciences, China-Japan Friendship Hospital, Beijing, China; 5 Institute of Respiratory Medicine, Chinese Academy of Medical Science, Beijing, China; 6 National Clinical Research Center of Respiratory Diseases, Beijing, China; 7 Laboratory of Clinical Microbiology and Infectious Diseases, China-Japan, Friendship Hospital, Beijing, China; 8 Department of Radiology, China-Japan Friendship Hospital, Beijing, China; 9 Clinical Center for Pulmonary Infections, Capital Medical University, Beijing, China; 10 Tsinghua University-Peking University Joint Center for Life Sciences, Beijing, China; 11 Chinese Academy of Medical Sciences and Peking Union Medical College, Beijing, China

**Keywords:** Bronchiectasis, NTM pulmonary disease, Clinical characteristics, Associated factors

## Abstract

**Background:**

The diagnosis and treatment of patients with bronchiectasis and nontuberculous mycobacterium (NTM) pulmonary disease are challenging issues and the treatment is also prolonged and depends on the species. There is limited information on patients with bronchiectasis and NTM pulmonary disease in Mainland China.

**Methods:**

This cross-sectional study was conducted at the China–Japan Friendship Hospital, Beijing, China. Those adult patients who met the diagnostic criteria for bronchiectasis and obtained a culture result of mycobacteria from lower respiratory tract specimens or lung tissue were included in this study. A logistic regression model was used to identify the related factors in patients with NTM pulmonary disease.

**Results:**

A total of 202 patients with bronchiectasis from 19 cities, 155 without and 47 (23.3%) with NTM pulmonary disease, were included. In all the 47 patients with NTM pulmonary disease, *Mycobacterium avium* complex was the most common species (66.0%), and 72.3% of them were initiated on standard anti-NTM treatment within 3 months after the diagnosis of NTM pulmonary disease. A larger proportion of patients with NTM pulmonary disease had acute exacerbations of ≥ 3 times within 1 year and were diagnosed bronchiectasis ≥ 50 years among patients with NTM pulmonary disease. The HRCT chest images revealed higher proportions of nodular shadow (100% vs. 35.3%), tree-in-bud sign (97.9% vs. 29.0%), cavities (29.8% vs. 5.8%), and airway dilation of the right middle lobe or the left lingular lobe (63.8% vs. 23.9%) in patients with NTM pulmonary disease than in those without NTM pulmonary disease (all P values = 0.001). The multivariable logistic regression model indicated that three and more abnormal features (OR 33.8; 95% CI 11.1–102.8) and main lesions of bronchial expansion in the middle or lingual lobe (OR 6.4; 95% CI 2.4–16.6) in HRCT chest images were independently associated with NTM pulmonary disease (P values = 0.001).

**Conclusion:**

In a single center of Mainland China, > 23% of patients with bronchiectasis had NTM pulmonary disease, and most patients were started on standard treatment within 3 months after the diagnosis of NTM pulmonary disease. These findings suggest that patients with bronchiectasis should be thoroughly examined for the presence of NTM pulmonary disease.

***Trial registration*:**

NCT03594032.

## Background

Bronchiectasis of noncystic fibrosis origin or noncystic fibrosis bronchiectasis is a chronic structural lung disease characterized by recurrent episodes of lung infection [1]. The colonization and infection of different pathogenic microorganisms can promote the formation of bronchiectasis and affect its severity and prognosis [2, 3]. Bronchiectasis accompanied with positive isolation of *Pseudomonas aeruginosa* has been associated with worsening lung function, increased frequencies of acute exacerbations, and poor prognosis [1, 4–6]. The isolation rate of viruses and new bacteria in the sputum of patients with acute exacerbation of bronchiectasis has also significantly increased [7]. Detection of *Aspergillus fumigatus* in the airways of patients with bronchiectasis is associated with risks for severe disease, worse lung function, and acute exacerbations [8]. Over the past 10 years, the issue of whether the nontuberculous mycobacterium (NTM) pulmonary disease was hospital-acquired or community-acquired had been an interesting research question in the clinical investigations of patients with bronchiectasis [9–13].

NTM, mycobacteria other than the *Mycobacterium tuberculosis* complex, and *M. leprae*, are widely distributed in natural environments. More than 190 species of NTM have been discovered, of which only a few are pathogenic and conditional pathogens [14]. The human infection of NTM was first reported among immunocompromised patients such as those infected with the human immunodeficiency virus (HIV) [15, 16]. The identified risk factors for NTM infection include structural lung disease, lung cancer, receiving immunosuppressive therapy, organ transplantation, HIV infection, and old age [16, 17]. The 5-year all-cause mortality of NTM pulmonary diseases is 10–45%, which is primarily caused by comorbidities, as the mortality related to NTM pulmonary disease is relatively low [17]. Currently, the prevalence rate of NTM pulmonary disease is uncertain, and studies have reported an obvious regional difference, although the prevalence rate has been increasing globally in recent years [18, 19]. In countries with a high burden of tuberculosis, the isolation rate of NTM has significantly increased in recent years [20–22]. Although the exact impact of NTM pulmonary disease on chronic lung disease remains unclear, patients with chronic obstructive pulmonary disease were found to have a greater risk for worsening situation and high mortality if they were NTM-positive [23, 24]. Patients with bronchiectasis accompanied with a positive result for the *Mycobacterium avium* complex (MAC) have a higher risk for death [9].

The relationship between bronchiectasis and NTM pulmonary infection is complicated. It is well known that NTM infection can cause bronchiectasis. In recent years, accumulating evidence has confirmed that NTM infection can cause severe bronchiectasis [25–27]. However, NTM pulmonary disease can present as nodular bronchiectasis on computed tomography (CT), which leads to a misdiagnosis of bronchiectasis with bacterial infection. The prevalence of NTM infection in patients with bronchiectasis is unclear. Previous studies have reported that the positive isolation rate of NTM in patients with bronchiectasis is 1.7–30% [9, 25], whereas the rate is up to 54% in US [28]. The diagnosis and treatment of NTM pulmonary disease still remain challenging issues in clinical practice. Recent guidelines recommend that the diagnosis of NTM pulmonary disease must be based on clinical manifestations and imaging and microbiological results [14]. However, patients with bronchiectasis always have respiratory symptoms during stable and acute exacerbations, and chest CT can also show nodules, bronchiectasis, and cavities caused by other pathogenic infections, causing difficulty in diagnosing NTM pulmonary disease in these patients. A positive result of NTM does not guarantee the diagnosis of the disease, and the diagnosis of NTM pulmonary disease is often delayed because the symptoms are mild and the excretion of NTM in the sputum is intermittent with few colonies retrievable in culture [17]. Furthermore, the proportions of misdiagnosis and inappropriate treatment of NTM are largely unknown [29]. However, there exists limited evidence in this regard, and especially, there are still no data from Mainland China to evaluate the clinical characteristics and other related characteristics among patients with bronchiectasis and NTM.

Therefore, we conducted this cross-sectional study to evaluate the prevalence, clinical characteristics, and treatment status of patients with NTM pulmonary disease, to evaluate their clinically predictive significance, and to provide evidence for the management of bronchiectasis with NTM pulmonary disease.

## Method

### Design and population

This cross-sectional study was conducted at the China–Japan Friendship Hospital in Beijing, China, from August 2018 to December 2020. Patients were included if they met the following inclusion criteria: (1) age ≥ 18 years, (2) diagnosed with bronchiectasis according to the 2010 British Thoracic Society guideline for non-CF bronchiectasis [30], and (3) at least two sputum or one bronchoalveolar lavage fluid (BALF) mycobacterial culture examination from 1 year before enrollment to 3 months after enrollment [31]. Patients were excluded if they met any one of the following exclusion criteria: (1) diagnosed with cystic fibrosis, (2) history of organ transplantation, (3) presence of active tumors or hematological malignancies, (4) diagnosed with pulmonary interstitial fibrosis with bronchiectasis, (5) diagnosed with allergic bronchopulmonary aspergillosis (ABPA), (6) diagnosed with diffuse panbronchiolitis, (7) excluded as NTM pulmonary disease based on clinical and radiological manifestations, despite positive NTM cultures, or (8) refusal of signed consent. All patients with bronchiectasis received standardized treatments according to ATS guidelines. This study was approved by the Ethics Committee of the China–Japan Friendship Hospital (ethics number: 2018-62-K46) and registered for the clinical trial (No. NCT03594032). All the included participants signed the informed consent.

Details regarding age, height, weight, smoking status, marital status, medical history, comorbidity, drug use, and treatment were self-reported and recorded. The characteristics of sputum among patients with previous acute exacerbations were also self-reported and recorded. All the included information and data were independently collected by two physicians separately and subjected to a consistency test to check the discrepancy of the recorded information.

Nontuberculous mycobacterial pulmonary disease (NTM-PD) was diagnosed according to the 2007 American Thoracic Society guideline for Nontuberculous Mycobacterial Lung Disease [31] and NTM pulmonary infection was diagnosed based on progressive or new abnormal images in HRCT.

The duration of bronchiectasis diagnosis was calculated as the duration between the time of enrollment (year) and the diagnosis time of bronchiectasis (year). The time of bronchiectasis diagnosis was defined as the time for the patient to be first diagnosed with bronchiectasis by physicians according to symptoms and imaging findings (chest CT or chest radiograph).

The etiology of bronchiectasis was determined if the patient met any one of the following conditions: (1) postinfection: a definitive history of tuberculosis or measles pneumonia or a history of hospitalization due to lower respiratory tract infection or a first diagnosis of bronchiectasis with NTM infection; (2)immunodeficiency: a decrease in blood immunoglobulin level confirmed by twice laboratory tests within 2 weeks; (3)idiopathic: without specific causes or without tests performed to screen for the causes; and (4) others: history of COPD or rheumatoid diseases.

The modified Medical Research Council (mMRC) dyspnea scale, the FACED score, and the bronchiectasis severity index were evaluated by professional physicians under the standard operation procedures. The mMRC dyspnea scale is a 5-point (0–4) scale used to evaluate dyspnea, disability, and functions, which is rated for the following five categories: grade 0/1 (patient does not experience dyspnea except on intensive exercise), grade 1/2 (getting short breath when hurrying on the ground level or walking up a slight slope), grade 2/3 (walking slower than most peers with similar age due to dyspnea or having to stop to breathe after walking 15 min on foot at own pace), grade 3/4 (stopping for breath after walking about 100 m or after a few minutes on the ground level), and grade 4/5 (being too breathless to leave the house or to undress themselves). The FACED score evaluates the severity and prognosis of noncystic fibrosis bronchiectasis according to the following five characteristics of patients [32]: lung function (FEV1% predicted), age, microbiological (chronic colonization by *P. aeruginosa*), radiological (number of infected lobes), and clinical syndromes (degree of dyspnea, appraised by the mMRC scale). Based on the total FACED score, we divided the patients into the following three grades: mild bronchiectasis (global score 0–2 points), moderate bronchiectasis (global score 3–4 points), and severe bronchiectasis (global score 5–7 points). The bronchiectasis severity index is a scale that evaluates the severity and prognosis of noncystic fibrosis bronchiectasis through the following nine related variables [32]: age, body mass index (BMI), predicted FEV1%, hospitalization and exacerbations before study, degree of dyspnea, chronic colonization by *P. aeruginosa* along with other microorganisms, and radiological extension.

Bronchiectasis was diagnosed based on the evaluation of chest high-resolution computed tomography (HRCT) images if any one of the following criteria was met [30]: (1) broncho-arterial ratio > 1 (internal airway lumen vs adjacent pulmonary artery), (2) lack of tapering, and (3) airway visibility within 1 cm of costal pleural surface or touching the mediastinal pleura. Chest HRCT images before the time of enrollment were collected. If the chest HRCT before the enrollment was unavailable, a new test was performed for these patients within 3 months after enrollment. And these CT images were evaluated by two radiologists independently according to the above-described criteria. Bronchiectasis lesion range was evaluated by semiquantitative estimation and divided into the following three categories: lesion range/lung lobes < 1/3, or 1/3–1/2, or ≥ 1/2. Abnormal imaging features were defined as those with three or more of the following characteristics simultaneously: consolidation/infiltration, nodular shadow, tree-in-bud sign, and cavity.

All microbiological examinations of specimens collected from the lower respiratory tract, including smears using standard methods, bacterial culture, fungal culture, acid-fast bacilli smears, and mycobacterial liquid culture, were conducted at the Clinical Microbiological Laboratory of the China–Japan Friendship Hospital, Beijing. The automated BACTEC MGIT 960 mycobacteria culture system was used, and the NTM species were identified by PCR-reverse dot blot.

### Statistical analyses

Continuous variables with normal distribution were expressed as mean ± standard deviation and analyzed by *t*-test for two independent samples. Categorical variables were expressed as frequency and percentages, and the chi-square test or Fisher’s exact test was used to compare the frequencies between two groups. Both univariate logistic regression models and multivariable logistic regression models were used to evaluate the relationship between the conditions of NTM. For the multivariable model, variables, including age, gender, BMI, age at diagnosis of bronchiectasis (≥ 50 and < 50 years), times of bronchiectasis exacerbations in the previous year (≥ 3 and < 3 times), HRCT-based bronchiectasis involving bilateral lungs, HRCT-based branch expansion main lesions in the middle or lingual lobe, and HRCT-based ≥ 3 abnormal imaging features, were selected and included into the model using a stepwise method.

All statistical analyses were conducted using the SPSS21 software (IBM, Armonk, New York). A two-tailed P value of < 0.05 was considered to be statistically significant.

## Results

### Participants

**Fig. 1:**
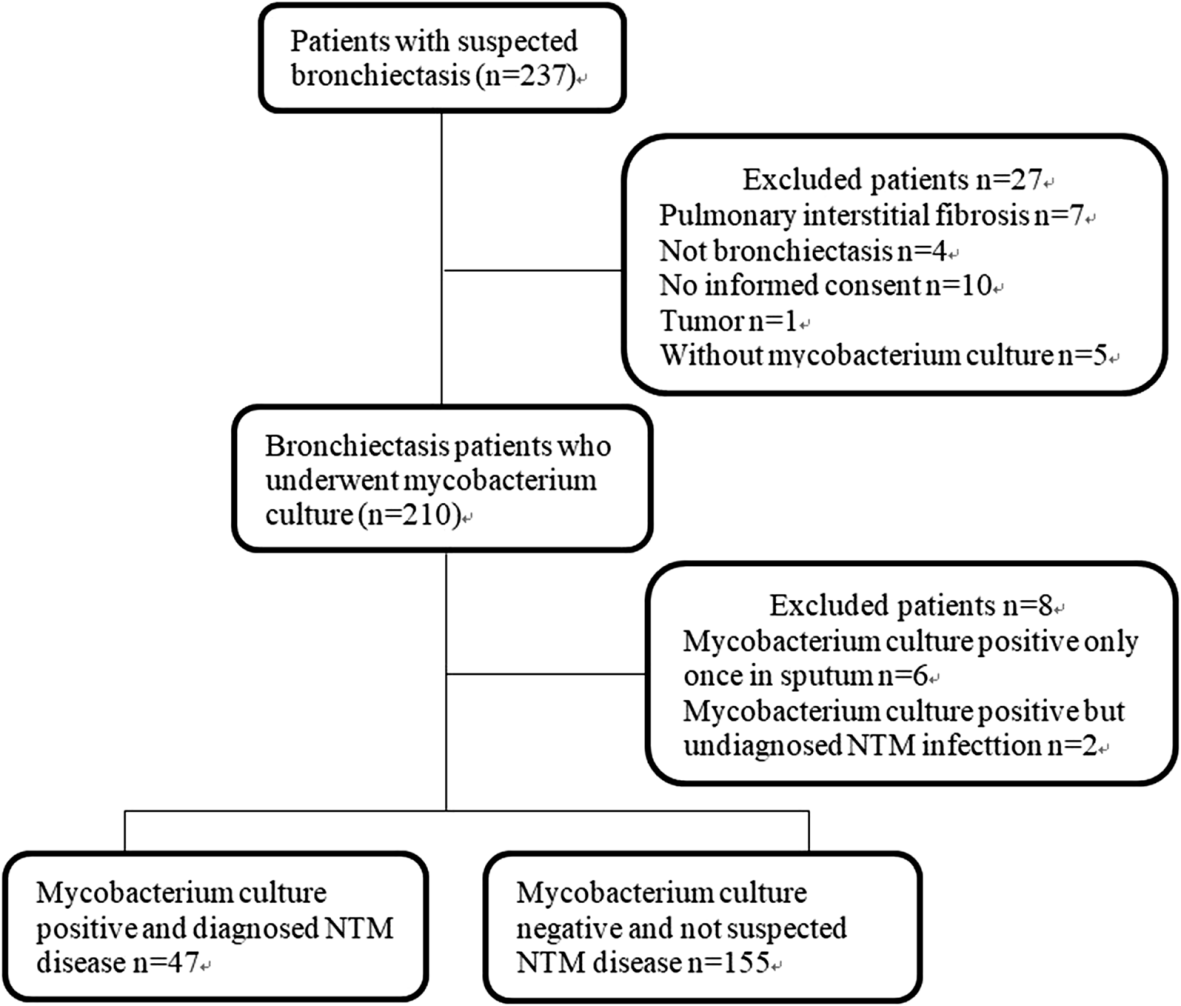
Study flowchart of patients with noncystic fibrosis bronchiectasis who underwent *Mycobacterium* culture. *NTM* nontuberculous mycobacterium disease

A total of 237 patients with suspected bronchiectasis were screened, of whom 202 patients with bronchiectasis were included in this study (Fig. 1). There were 136 (67.3%) hospitalized patients, 115 patients (56.9%) from Beijing and 87 patients (43.1%) from 18 provinces of Mainland China. Among them, 47 patients aged 32–81 years were diagnosed with bronchiectasis combined with NTM pulmonary disease, and 89.2% of these patients were women with menopause. Among those patients, 29 (61.7%) were from Beijing, and the remaining 18 (38.3%) were from 12 provinces of China. In total, 23.3% of the patients were diagnosed with NTM diseases, and 72.3% (34/47) were started on standard treatment within 3 months after the diagnosis of NTM pulmonary disease (Initial antimicrobial therapy: Clarithromycin, rifampicin and ethambutol in patients with MAC-PD or M. kansasii –PD; Amikacin, Cefoxitin, macrolide and fluoroquinolone in patients with *M. abscessus*/*M. chelonae*-PD). MAC comprised the most common species (66.0%), followed by the *M. abscessus*/*M. chelonae* complex (27.6%) and then *M. kansasii* (6.4%) (Table 1). There were 68.1% of patients with positive isolation of NTM from BALF or lung tissue. Only 17% of these patients showed a positive result on acid-fast bacilli smear from the sputum or BALF. The most common symptom was cough or sputum (89.4%), fever was observed among only 6.4% of patients with NTM, and only two patients had chest pain. The chest HRCT findings of the 47 patients with bronchiectasis with nodules/tree-in-bud sign showed that 72.3% of them had non-centrilobular nodules and only 29.8% had cavity. A positive result on the interferon-gamma release assay was found in18.6% of patients (Table 1).

**Table 1:** Characteristics of 47 bronchiectasis patients with NTM pulmonary disease

Characteristics	Cases (%) n = 47
Age at diagnosis of NTM pulmonary disease, years, mean ± SD (range)	62.0 ± 10.6 (32–81)
Menopause	33/37 (89.2)
Species of NTM isolation	
*M. avium* complex (MAC)	31 (66.0)
*M. abscessus*/*M. chelonae*	13 (27.6)
*M. kansasii*	3 (6.4)
Specimens and frequency of NTM positive isolation	
≥ 2 times of NTM positive isolation from sputum	15 (31.9)
NTM positive isolation from BALF or lung tissue	32 (68.1)
Positive acid-fast bacilli smear from sputum/BALF	8 (17.0)
Symptoms at diagnosis of NTM pulmonary disease	
Cough/expectorant	42 (89.4)
Hemoptysis	12 (25.0)
Short of breath	15 (31.9)
Fever	3 (6.4)
Chest pain	2 (4.3)
Chest HRCT imaging feature of	
Bronchiectasis with nodules/tree-in-bud	47 (100)
Non-centrilobular nodules	34 (72.3)
Cavity	14 (29.8)
IGRA	
Positive	8/43 (18.6)
Treatment*	34/47 (72.3)

*BALF* bronchoalveolar lavage fluid; *HRCT* high-resolution computed tomography; *IGRA* interferon-gamma release assay

*Treatment: Prescribed three or more drugs against NTM within 3 months when NTM pulmonary disease (PD) was diagnosed by a physician. Initial antimicrobial therapy: Clarithromycin, rifampicin and ethambutol in patients with MAC-PD or M. kansasii–PD; Amikacin, Cefoxitin, macrolide and fluoroquinolone in patients with *M. abscessus*/*M. chelonae*-PD

### Demographic characteristics among patients with or without NTM pulmonary disease

The majority of demographic characteristics, including smoking status, past medical history, and most comorbidities, were similar between patients with NTM infection and those without NTM infection (all P values > 0.05) (Table 2). Compared with patients without NTM pulmonary disease, those with NTM pulmonary disease were slightly older in age (P = 0.042), more likely to be female (P = 0.023), more likely to have low BMI levels (P = 0.007), and had less incidence of cardiovascular diseases (P = 0.028) and diabetes (P = 0.004).

**Table 2:** Demographic characteristics of bronchiectasis patients with and without NTM pulmonary disease

Characteristic	Patients with NTM	Patients without NTM	*P* value
No. of patients	47	155	
Specimens of NTM isolation			
BALF	30 (63.8)	85 (54.8)	**0.276**
Age (years) mean ± SD	62.6 ± 10.7	58 ± 14	**0.042**
Female n (%)	37 (78.7)	96 (60.6)	**0.023**
BMI < 20 kg/m^2^	23 (48.9)	43 (27.7)	**0.007**
Smoking status: n (%)			0.125
Current	5 (10.6)	12 (7.7)	
Second-hand smoke	4 (8.5)	16 (10.3)	
Past	2 (4.3)	26 (16.8)	
Never	36 (76.6)	101 (65.2)	
Selected comorbidities^a^			
Cardiovascular	10 (21.3)	60 (38.7)	**0.028**
Cerebrovascular	2 (4.3)	11 (7.1)	0.737
Osteoporosis	3 (6.4)	10 (6.5)	1.000
Depression	1 (2.1)	2 (1.3)	0.550
Diabetes	1 (2.1)	30 (19.4)	**0.004**
Asthma	2 (4.3)	5 (3.2)	0.665
COPD	4 (8.5)	23 (14.8)	0.264
Sinusitis	3 (6.4)	19 (12.3)	0.257
Prior medical history^a^			
Tuberculosis	11 (23.4)	27 (17.4)	0.358
Connective tissue disease	1 (2.1)	12 (7.7)	0.307
Gastro-esophageal reflux disease	8 (17.0)	17 (11.0)	0.270
Tumor	3 (6.4)	6 (3.9)	0.437
Lobectomy	2 (4.3)	13 (8.4)	0.528

*BMI* Body Weight Index; *COPD* Chronic obstructive pulmonary disease; *BALF* bronchoalveolar lavage fluid

^a^Comorbidities and prior medical history were extracted from clinical case data or patient answers

### Clinical manifestations of patients with or without NTM pulmonary disease

Clinical manifestations, including the disease course of bronchiectasis, mMRC scores, and the severity of bronchiectasis, were similar between patients with and without NTM pulmonary disease (all *P* values > 0.05). Compared with non-NTM infectious patients, a greater proportion of patients with NTM infection tended to have acute exacerbations of ≥ 3 times within 1 year (61.7% vs. 36.1%; P = 0.002). Those with NTM infection tended to be of older age with a diagnosis of bronchiectasis, and based on a diagnosis age of ≥ 50 years, there were 74.5% of patients with NTM and 56.8% of patients without NTM (P = 0.029). The results of spirometry showed that 185 patients (91.6%) had lung function results (FEV1%) within 6 months of enrollment, whereas a nonstatistical difference was observed between patients with NTM pulmonary disease and those without NTM pulmonary disease (Table 3).

**Table 3:** Clinical characteristics of bronchiectasis patients with and without NTM pulmonary disease

Characteristic	Patients with NTM	Patients without NTM	*P* value
No. of patients	47	155	
Course of bronchiectasis, n (%)			0.290
< 1 year	17(36.2)	44(28.4)	
1–10 years	20 (42.5)	60 (38.7)	
≥ 10 years	10 (21.3)	51 (32.9)	
Age at diagnosis of bronchiectasis ≥ 50 years	35 (74.5)	88 (56.8)	**0.029**
Etiology of bronchiectasis, n (%)			**0.001**
Post-infective	36 (76.6)	59 (38.1)	
Idiopathic	9 (19.1)	74 (47.7)	
Others	2 (4.3)	22 (14.2)	
COPD	2 (4.3)	13 (8.4)	
Rheumatoid arthritis	0 (0)	6 (3.9)	
Immunodeficiency	0 (0)	3 (1.9)	
mMRC ≤ 2 scores	43 (91.5)	128 (82.6)	0.138
FACED # n (%)			0.901
Mild	30 (66.7)	98 (70.0)	
Moderate	10 (22.2)	29 (20.7)	
Sever	5 (11.1)	13 (9.3)	
BSI # n (%)			0.130
Low	8 (17.8)	21 (15.0)	
Moderate	8 (17.8)	47 (33.6)	
High	29 (64.4)	72 (51.4)	
≥ 3 times of bronchiectasis exacerbations in last year	29 (61.7)	56 (36.1)	**0.002**
≥ 1 times of in-hospital in last 2 years	35 (74.5)	119 (76.8)	0.745
Microbial culture results, n (%)			
*Pseudomonas aeruginosa* positive isolation	11 (23.4)	37 (24.0)	0.930
Other bacteria positive isolation	7 (14.9)	34 (21.9)	0.313
Fungus positive isolation	6 (12.8)	16 (10.3)	0.789
Chest HRCT imaging, n (%)			
Bronchiectasis involved bilateral lungs	39 (83.0)	105 (67.7)	**0.043**
Bronchiectasis involved ≥ 2 lung lobes	43 (91.5)	127 (81.9)	0.116
Bronchiectasis type			0.216
Cylindrical	35 (74.5)	94 (60.7)	
Cystic	1 (2.1))	7 (4.5)	
Mixed	11 (23.4)	54 (34.8)	
Bronchiectasis main lesions			
In upper lobe	6 (12.8)	24 (15.5)	0.646
In middle lobe or lingual	30 (63.8)	37 (23.9)	**0.001**
In lower lobe	17 (36.2)	110 (71.0)	**0.001**
With ≥ 3 abnormal imaging features	42 (89.4)	33 (21.3)	**0.001**
With consolidation/infiltrates shadow	43 (91.5)	127 (81.9)	0.116
With nodular shadow	47 (100)	55 (35.5)	**0.001**
With tree-in bud sign	46 (97.9)	45 (29.0)	**0.001**
With cavities	14 (29.8)	9 (5.8)	**0.001**
With swollen lymph nodes in mediastinum	8 (17.0)	25 (16.1)	0.885
FEV1 (% predicted)^#^			0.379
FEV1 ≥ 80%	26 (57.8)	65 (46.4)	
50 ≤ FEV1 < 80%	12 (26.7)	43 (30.7)	
FEV1 < 50%	7 (15.5)	32 (22.9)	

*COPD* chronic obstructive pulmonary disease; *BSI* bronchiectasis severity index. (low: 0–4 scores; moderate: 5–8 scores; high: 9–26 scores); FACED: (mild: 0–2 scores; moderate: 3–4 scores; severity: 5–7 scores): ^#^No. of patients: 185

### Microbiological evaluation

Of all the 202 patients, 115 had BALF specimens (30 with NTM infection and 85 without NTM infection), and among the 30 patients with NTM infection, 8 did not provide sputum specimens. The proportion of BALF specimens were similar between patients with and without NTM pulmonary disease(63.8% vs. 54.8%, P > 0.05), Among all positive patients, 20 showed NTM-positive results in both sputum and BALF specimens, and 2 showed NTM-negative results in sputum specimens but NTM-positive results in BALF specimens. The specimens collected from the lower respiratory tract of all patients were examined using bacterial and fungal cultures. The positive isolation rate of *P. aeruginosa* was similar between patients with NTM infection and those without NTM infection (23.4% vs. 24.0%, P > 0.05), and the positive isolation rates of other bacteria and fungi were also similar (Table 3).

### Imaging measurements

Patients with NTM pulmonary disease showed higher proportions of nodular shadow (100% vs. 35.3%), tree-in-bud sign (97.9% vs. 29.0%), and cavities (29.8% vs. 5.8%) and more than three abnormal imaging features (89.4% vs. 21.3%) than those without NTM pulmonary disease (all P values = 0.001). Patients with NTM pulmonary disease had greater proportion of airway dilation of the right middle lobe or the left lingular lobe (63.8% vs. 23.9%, P = 0.001) and less proportion of lower lobe airway dilation (36.2% vs. 71.0%, P = 0.001) (Table 3).

### Associated factors of bronchiectasis with NTM pulmonary disease

We used univariable and multivariable logistic regression models to evaluate the possible associated factors and found that more than three thoracic HRCT abnormalities of imaging features and bronchiectasis main lesions in the middle or lingual lobe were independently associated with NTM pulmonary disease among all patients with bronchiectasis (both P values = 0.001), and their ORs were 33.8 (95% CI 11.1–102.8) and 6.4 (2.4–16.6), respectively (Table 4). Age, gender, BMI ≤ 20 kg/m^2^, a diagnosis age of ≥ 50 years of bronchiectasis, bronchiectasis involving bilateral lungs on HRCT, and acute exacerbations of ≥ 3 times within 1 year were significantly related to NTM pulmonary disease in the univariate logistic model (all P values < 0.05) but not in the multivariable logistic regression model (all P values > 0.05).

**Table 4:** Univariate and multivariate logistic regression analysis of predictive factors related to bronchiectasis patients with NTM pulmonary infection

Characteristics	Univariate logistic regression model		Multivariate logistic regression model		
	P	OR	P	OR	95%CI
Age	0.044	1.028	0.471	1.480	0.510–4.291
Gender	0.026	2.401	0.577	1.331	0.488–3.634
BMI ≤ 20 (kg/m^2^)	0.008	2.496	0.381	1.528	0.592–3.939
Age at diagnosis of bronchiectasis ≥ 50 years	0.032	2.221	0.066	2.583	0.939–7.107
≥ 3 times of bronchiectasis exacerbations in last year	0.002	2.848	0.060	2.499	0.962–6.496
HRCT Bronchiectasis involved bilateral lungs	0.047	2.321	0.177	0.417	0.117–1.483
HRCT bronchiectasis main lesions in middle lobe or lingual lobe	0.001	5.628	0.001	6.379	2.448–16.619
HRCT ≥ 3 abnormal image features	0.001	31.055	0.001	33.783	11.107–102.758

Considering the high proportion of patients with combined infections of both NTM and *P. aeruginosa*, we further conducted a sensitivity analysis after excluding patients with combined infections of NTM and *P. aeruginosa* and found that the results were similar (Table 5).

**Table 5:** Univariate and multivariate logistic regression analysis of predictive factors related to bronchiectasis patients with NTM pulmonary infection among patients without combination infection of *Pseudomonas aeruginosa*

Characteristics	Univariate logistic regression model		Multivariate logistic regression model		
	P	OR	P	OR	95% CI
Age	0.097	1.025	–	–	
Gender	0.060	2.239	–	–	
BMI ≤ 20 (kg/m^2^)	0.001	3.625	–	–	
Age at diagnosis of bronchiectasis ≥ 50 years	0.018	2.985	0.078	3.109	0.882–10.959
≥ 3 times of bronchiectasis exacerbations in last year	0.023	2.417	0.187	2.418	0.690–6.684
HRCT Bronchiectasis involved bilateral lungs	0.093	2.110	–	–	
HRCT bronchiectasis Main lesions in middle lobe or lingual lobe	0.001	6.667	0.001	7.641	2.417–24.158
HRCT ≥ 3 abnormal image features	0.001	30.07	0.001	32.754	9.669–110.961

## Discussion

This study was conducted on 202 patients with bronchiectasis from 19 provinces of China. Most patients had mild disease according to the FACED score, with a course of bronchiectasis of < 10 years. In total, 23.3% of patients were diagnosed with NTM pulmonary disease, and the *M. avium*—intracellular complex and *M. abscessus* branch complex were the primary pathogens isolated from their specimens. Unlike the majority of previous studies [9, 33–35] where only once-positive result of sputum specimen was used for NTM diagnosis, in the present study, all the 47 patients with NTM pulmonary disease fulfilled the criteria of NTM pulmonary disease and were diagnosed with NTM pulmonary infection based on progressive or new abnormal images in HRCT, and 72.3% of them were started on the standard treatment of anti-NTM within 3 months after diagnosis. Most clinical characteristics of patients with bronchiectasis with NTM pulmonary disease and those of patients without NTM pulmonary disease were similar. The multiple regression analysis revealed that HRCT imaging characteristics comprised the major difference between those with NTM pulmonary disease and without NTM pulmonary disease.

The HRCT manifestations of patients with NTM pulmonary disease and normal immune are generally divided into the following three types: fibrocavitary lesions, nodular bronchiectasis and hypersensitivity pneumonia [36]. Our study demonstrated that all patients with NTM pulmonary disease have the imaging characteristics of “nodular bronchiectasis” on HRCT, irrespective of the type of NTM, accompanied with tree-in-bud sign, consolidation shadow, infiltration shadow, and cavity. Similarly, a retrospective study reviewed the HRCT images of 29 patients with a positive bronchial wash culture result and found that the presence of bronchiectasis, cavitary nodules with feeding bronchus sign, and tree-in-bud nodules in the middle lobe and lingula are suggestive of NTM infection [37]. This central lobular nodule may indicate the spread of NTM infection along the bronchus [38]. We observed that 72.3% of patients with bronchiectasis and NTM pulmonary disease had nonlobular central nodules on HRCT. Another study [39] evaluated the thoracic HRCT images of 63 patients with NTM infection and reported that 81% had nodules, 39.8% had lobular centrality, 11.6% had peripheral lymphatics, 9.5% had random distribution, and 20.6% had mixed. NTM are less pathogenic than tuberculous bacteria; hence, compared with tuberculosis, NTM pulmonary disease has fewer cavities [40] and thinner wall [36]. In our study, 29.8% of NTM cases had cavitation on HRCT, which was significantly higher than that in patients with non-NTM pulmonary disease (5.8%). In patients with bronchiectasis combined with NTM pulmonary disease, the lesions were primarily located in the middle or lingual lobe and were independently related to the NTM pulmonary disease. However, in patients without NTM pulmonary disease, the lesions were primarily located in the lower lobe. These results were consistent with previous findings [28]. Although imaging characteristics can be used to distinguish NTM infection from bronchiectasis caused by common bacterial infections and are helpful for the early diagnosis of NTM infection, the major concern is the lack of specificity. Lung infections caused by other pathogens, such as fungal infection, might present similar radiological findings. We thought the dynamic changes of images and the typical image characteristics of NTM pulmonary disease might help physician to clinically diagnose NTM pulmonary disease. Nevertheless, it is necessary to evaluate and incorporate clinical systemic inflammatory responses and microbial testing to aid in the differential diagnosis.

Unlike tuberculosis, NTM pulmonary infection does not induce specific clinical symptoms in hosts with normal immunity [41]. Consistent with previous studies [28, 42], our study demonstrated that patients with bronchiectasis and NTM pulmonary infection rarely have fever. We did not detect a significant difference in the culture positive rate of *P. aeruginosa*, the course of bronchiectasis, the severity of bronchiectasis, and lung function FEV1% in patients with and without NTM pulmonary disease, which is consistent with previous studies [11, 34]. The study of Sulaiman [43] elucidated the difference in the sputum microbiome between patients with bronchiectasis with or without NTM pulmonary disease, which might support that there is a lack of special clinical characteristics in these patients with NTM pulmonary disease. Studies have reported that NTM pulmonary disease is more likely to occur among low-weight postmenopausal women [16, 33, 44], and our study also suggested that patients with bronchiectasis and NTM pulmonary disease tended to be of low weight and older aged women, as 74.5% cases of NTM pulmonary disease were diagnosed at the age of > 50 years. Compared with patients without NTM, a greater number of patients with NTM pulmonary disease frequently had acute exacerbations in the past year, which is different from previous studies. A study conducted in US evaluated 1826 patients and observed fewer pulmonary exacerbations in the past 2 years among patients with NTM infection than those among patients without NTM infection [28]. The discrepancy in these results may be due to the heterogeneity of characteristics of the study population, such as comorbidities, severity of bronchiectasis, and combination with *P. aeruginosa* infection. NTM disease is a chronic infection, as studies have demonstrated that 41.2%–55.3% of patients with nodular bronchiectasis and NTM pulmonary disease show worsening HRCT findings for a follow-up of 10 years [45, 46]. Our data also suggested NTM infection is a risk factor for the aggravation of bronchiectasis. However, the impact of NTM infection on the prognosis of patients with bronchiectasis is worthy of a thorough investigation in the future.

In this study, the most commonly isolated NTM in patients with bronchiectasis was MAC, and the least commonly isolated NTM was *M. abscessus*/*M. chelonae*, which was consistent with previous studies [9, 47–49]. In general, the species of NTM isolated from the lower respiratory tract differ according to regions and countries. For instance, the primary bacterium causing NTM disease was the *M. abscessus* branch complex in Guangzhou [50] and the MAC in Beijing [22, 51]. In the present study population, the primary bacterium causing NTM disease was the MAC, which might because most patients were from Beijing. Although MAC has been predominantly isolated in most countries from pulmonary samples, *M. abscessus*/*M. chelonae* was found to be the predominant pathogenic species in Singapore [42]. Furthermore, *M. xenopi* was the most frequently isolated species, followed by MAC, in Southern Europe [52]. The prevalence of NTM diseases was 23.3%, which is higher than that reported by a meta-analysis (9.3%) [53]. This might be because the patients included in this study were the refractory patients nationally, who were difficult to manage than the general bronchiectasis population in Mainland China, and we used strict criteria for the diagnosis. The specimens used in this study for mycobacterial examination were BALF or lung tissue, and the proportion of BALF specimens were similar between patients with and without NTM pulmonary disease,which might largely improve the sensitivity and accuracy of diagnosis.

Our study has some limitations. First, it was a single-center study conducted on a small number of participants, which may limit the generalizability of our conclusion. However, the patients were from 19 provinces of China. Second, the residual confounders such as the treatment of bronchiectasis and NTM infection cannot be excluded. Third, because of the cross-sectional design of this study, it is difficult to reach a causal conclusion. We collected numerous clinical data and characteristics of patients with bronchiectasis in the present study to systematically evaluate the prevalence, clinical characteristics, and treatment status of patients with NTM pulmonary disease. Nevertheless, due to the study limitations, further well-designed prospective studies with a large sample size are required to further determine the impact of NTM pulmonary disease on the severity of the disease and the prognosis of patients with bronchiectasis.

## Conclusions

In the present study patient population, the prevalence of NTM pulmonary disease among those with bronchiectasis was 23.3%, and the majority of patients (72.3%) were initiated on standard anti-NTM treatment. Nodular bronchiectasis was identified as the primary feature on the HRCT images of patients with bronchiectasis with NTM pulmonary disease. Chest HRCT scans revealed that three and more abnormal imaging features (consolidation/infiltration, nodules, tree-in-buds, and cavity) or bronchiectasis main lesions in the middle or lingual lobe were independently associated with NTM pulmonary disease. Our findings suggest that it is necessary to clearly consider the presence of NTM pulmonary disease and better management strategies in patients with bronchiectasis.

## Data Availability

The datasets used and/or analyzed during the current study are available from the corresponding author on reasonable request.

## Abbreviations

NTM: Nontuberculous mycobacterium
HRCT: High-resolution computed tomography
HIV: Human immunodeficiency virus
MAC: *Mycobacterium avium* complex
ABPA: Allergic bronchopulmonary aspergillosis
BMI: Body mass index

## References

[1] McShane PJ, Tino G (2019). Bronchiectasis. Chest.

[2] Rogers GB, van der Gast CJ, Cuthbertson L, Thomson SK, Bruce KD, Martin ML, Serisier DJ (2013). Clinical measures of disease in adult non-CF bronchiectasis correlate with airway microbiota composition. Thorax.

[3] Dickson RP, Martinez FJ, Huffnagle GB (2014). The role of the microbiome in exacerbations of chronic lung diseases. Lancet (London, England).

[4] Polverino E, Goeminne PC, McDonnell MJ, Aliberti S, Marshall SE, Loebinger MR, Murris M, Cantón R, Torres A, Dimakou K (2017). European Respiratory Society guidelines for the management of adult bronchiectasis. Eur Respir J.

[5] Kamata H, Asakura T, Suzuki S, Namkoong H, Yagi K, Funatsu Y, Okamori S, Uno S, Uwamino Y, Fujiwara H (2017). Impact of chronic *Pseudomonas aeruginosa* infection on health-related quality of life in *Mycobacterium avium* complex lung disease. BMC Pulm Med.

[6] Finch S, McDonnell MJ, Abo-Leyah H, Aliberti S, Chalmers JD (2015). A comprehensive analysis of the impact of *Pseudomonas aeruginosa* colonization on prognosis in adult bronchiectasis. Ann Am Thorac Soc.

[7] Chen CL, Huang Y, Yuan JJ, Li HM, Han XR, Martinez-Garcia MA, de la Rosa-Carrillo D, Chen RC, Guan WJ, Zhong NS (2020). The roles of bacteria and viruses in bronchiectasis exacerbation: a prospective study. Arch Bronconeumol.

[8] Mac Aogáin M, Chandrasekaran R, Lim AYH, Low TB, Tan GL, Hassan T, Ong TH, Hui Qi Ng A, Bertrand D, Koh JY (2018). Immunological corollary of the pulmonary mycobiome in bronchiectasis: the CAMEB study. Eur Respir J.

[9] Shteinberg M, Stein N, Adir Y, Ken-Dror S, Shitrit D, Bendayan D, Fuks L, Saliba W (2018). Prevalence, risk factors and prognosis of nontuberculous mycobacterial infection among people with bronchiectasis: a population survey. Eur Respir J.

[10] Griffith DE, Aksamit TR (2018). *Mycobacterium avium* complex and bronchiectasis there's something happening here. Am J Respir Critic Care Med.

[11] Fowler SJ, French J, Screaton NJ, Foweraker J, Condliffe A, Haworth CS, Exley AR, Bilton D (2006). Nontuberculous mycobacteria in bronchiectasis: prevalence and patient characteristics. Eur Respir J.

[12] Park IK, Olivier KN (2015). Nontuberculous mycobacteria in cystic fibrosis and non-cystic fibrosis bronchiectasis. Semin Respir Crit Care Med.

[13] Piersimoni C, Scarparo C (2008). Pulmonary infections associated with non-tuberculous mycobacteria in immunocompetent patients. Lancet Infect Dis.

[14] Daley CL, Iaccarino JM, Lange C, Cambau E, Wallace RJ, Andrejak C, Böttger EC, Brozek J, Griffith DE, Guglielmetti L (2020). Treatment of nontuberculous mycobacterial pulmonary disease: an official ATS/ERS/ESCMID/IDSA clinical practice guideline. Eur Respir J.

[15] Griffith DE (2003). Emergence of nontuberculous mycobacteria as pathogens in cystic fibrosis. Am J Respir Crit Care Med.

[16] Cowman S, van Ingen J, Griffith DE, Loebinger MR (2019). Non-tuberculous mycobacterial pulmonary disease. Eur Respir J.

[17] Larsson LO, Polverino E, Hoefsloot W, Codecasa LR, Diel R, Jenkins SG, Loebinger MR (2017). Pulmonary disease by non-tuberculous mycobacteria - clinical management, unmet needs and future perspectives. Expert Rev Respir Med.

[18] Kim HO, Lee K, Choi HK, Ha S, Lee SM, Seo GH (2019). Incidence, comorbidities, and treatment patterns of nontuberculous mycobacterial infection in South Korea. Medicine.

[19] Axson EL, Bual N, Bloom CI, Quint JK (2019). Risk factors and secondary care utilisation in a primary care population with non-tuberculous mycobacterial disease in the UK. Eur J Clin Microbiol Infect Dis.

[20] Xu D, Han C, Wang MS, Wang JL (2018). Increasing prevalence of non-tuberculous mycobacterial infection from 2004–2009 to 2012–2017: a laboratory-based surveillance in China. J Infect.

[21] Xu J, Li P, Zheng S, Shu W, Pang Y (2019). Prevalence and risk factors of pulmonary nontuberculous mycobacterial infections in the Zhejiang Province of China. Epidemiol Infect.

[22] Li YM, Tong XL, Xu HT, Ju Y, Cai M, Wang C (2016). Prevalence and antimicrobial susceptibility of *Mycobacterium abscessus* in a General Hospital, China. Biomed Environ Sci.

[23] Wyrostkiewicz D, Szturmowicz M, Bartoszuk I, Siemion-Szczesniak I, Jakubowska L, Augustynowicz-Kopec E, Kus J (2018). Nontuberculous mycobacterial lung disease in a patient with COPD and bronchiectasis, with radiological signs of lung tumor. Adv Respir Med.

[24] Sun YX, Shao CK, Li S, Xu K, Huang H, Xu ZJ (2019). The clinical analysis of chronic obstructive pulmonary disease patients complicated with nontuberculous mycobacterial pulmonary disease. Zhonghua jie he he hu xi za zhi Zhonghua jiehe he huxi zazhi Chinese journal of tuberculosis and respiratory diseases.

[25] Bonaiti G, Pesci A, Marruchella A, Lapadula G, Gori A, Aliberti S (2015). Nontuberculous mycobacteria in noncystic fibrosis bronchiectasis. Biomed Res Int.

[26] Lam DL, Kapnadak SG, Godwin JD, Kicska GA, Aitken ML, Pipavath SN (2018). Radiologic computed tomography features of *Mycobacterium abscessus* in cystic fibrosis. Clin Respir J.

[27] Kimizuka Y, Hoshino Y, Nishimura T, Asami T, Sakakibara Y, Morimoto K, Maeda S, Nakata N, Abe T, Uno S (2019). Retrospective evaluation of natural course in mild cases of *Mycobacterium avium* complex pulmonary disease. PLoS ONE.

[28] Aksamit TR, O'Donnell AE, Barker A, Olivier KN, Winthrop KL, Daniels MLA, Johnson M, Eden E, Griffith D, Knowles M (2017). Adult patients with bronchiectasis: a first look at the US bronchiectasis research registry. Chest.

[29] Gill LI, Dominic C, Tiberi S (2021). Atypical mycobacterial infections - management - when to treat. Curr Opin Pulm Med.

[30] Pasteur MC, Bilton D, Hill AT (2010). British Thoracic Society guideline for non-CF bronchiectasis. Thorax.

[31] Griffith DE, Aksamit T, Brown-Elliott BA, Catanzaro A, Daley C, Gordin F, Holland SM, Horsburgh R, Huitt G, Iademarco MF (2007). An official ATS/IDSA statement: diagnosis, treatment, and prevention of nontuberculous mycobacterial diseases. Am J Respir Crit Care Med.

[32] Ellis HC, Cowman S, Fernandes M, Wilson R, Loebinger MR (2016). Predicting mortality in bronchiectasis using bronchiectasis severity index and FACED scores: a 19-year cohort study. Eur Respir J.

[33] Mirsaeidi M, Hadid W, Ericsoussi B, Rodgers D, Sadikot RT (2013). Non-tuberculous mycobacterial disease is common in patients with non-cystic fibrosis bronchiectasis. Int J Infect Dis.

[34] Faverio P, Stainer A, Bonaiti G, Zucchetti SC, Simonetta E, Lapadula G, Marruchella A, Gori A, Blasi F, Codecasa L (2016). Characterizing non-tuberculous mycobacteria infection in bronchiectasis. Int J Mol Sci.

[35] Máiz L, Girón R, Olveira C, Vendrell M, Nieto R, Martínez-García MA (2016). Prevalence and factors associated with nontuberculous mycobacteria in non-cystic fibrosis bronchiectasis: a multicenter observational study. BMC Infect Dis.

[36] Anjos L, Parreira P, Torres P, Kipnis A, Junqueira-Kipnis A, Rabahi M (2020). Non-tuberculous mycobacterial lung disease: a brief review focusing on radiological findings. Rev Soc Bras Med Trop.

[37] Polverosi R, Guarise A, Balestro E, Carloni A, Dalpiaz G, Feragalli B (2010). High-resolution CT of nontuberculous mycobacteria pulmonary infection in immunocompetent, non-HIV-positive patients. Radiol Med (Torino).

[38] Fujita J, Ohtsuki Y, Shigeto E, Suemitsu I, Yamadori I, Bandoh S, Shiode M, Nishimura K, Hirayama T, Matsushima T (2003). Pathological findings of bronchiectases caused by Mycobacterium avium intracellulare complex. Respir Med.

[39] Marušić A, Kuhtić I, Mažuranić I, Janković M, Glodić G, Sabol I, Stanić L (2020). Nodular distribution pattern on chest computed tomography (CT) in patients diagnosed with nontuberculous mycobacteria (NTM) infections. Wien Klin Wochenschr.

[40] Kendall BA, Varley CD, Choi D, Cassidy PM, Hedberg K, Ware MA, Winthrop KL (2011). Distinguishing tuberculosis from nontuberculous mycobacteria lung disease, Oregon, USA. Emerg Infect Dis.

[41] Holt MR, Kasperbauer SH, Koelsch TL, Daley CL (2019). Similar characteristics of nontuberculous mycobacterial pulmonary disease in men and women. Eur Respir J.

[42] Zhang ZX, Cherng BPZ, Sng LH, Tan YE (2019). Clinical and microbiological characteristics of non-tuberculous mycobacteria diseases in Singapore with a focus on pulmonary disease, 2012–2016. BMC Infect Dis.

[43] Sulaiman I, Wu BG, Li Y, Scott AS, Malecha P, Scaglione B, Wang J, Basavaraj A, Chung S, Bantis K (2018). Evaluation of the airway microbiome in nontuberculous mycobacteria disease. Eur Respir J.

[44] Henkle E, Aksamit TR, Barker AF, Curtis JR, Daley CL, Anne Daniels ML, DiMango A, Eden E, Fennelly K, Griffith DE (2017). Pharmacotherapy for non-cystic fibrosis bronchiectasis: results from an NTM Info & research patient survey and the bronchiectasis and NTM Research Registry. Chest.

[45] Kitada S, Uenami T, Yoshimura K, Tateishi Y, Miki K, Miki M, Hashimoto H, Fujikawa T, Mori M, Matsuura K (2012). Long-term radiographic outcome of nodular bronchiectatic *Mycobacterium avium* complex pulmonary disease. Int J Tuberc Lung Dis.

[46] Gochi M, Takayanagi N, Kanauchi T, Ishiguro T, Yanagisawa T, Sugita Y (2015). Retrospective study of the predictors of mortality and radiographic deterioration in 782 patients with nodular/bronchiectatic *Mycobacterium avium* complex lung disease. BMJ Open.

[47] Hu C, Huang L, Cai M, Wang W, Shi X, Chen W (2019). Characterization of non-tuberculous mycobacterial pulmonary disease in Nanjing district of China. BMC Infect Dis.

[48] Jhun BW, Moon SM, Jeon K, Kwon OJ, Yoo H, Carriere KC, Huh HJ, Lee NY, Shin SJ, Daley CL (2020). Prognostic factors associated with long-term mortality in 1445 patients with nontuberculous mycobacterial pulmonary disease: a 15-year follow-up study. Eur Respir J.

[49] Boyton RJ, Altmann DM (2016). Bronchiectasis: current concepts in pathogenesis, immunology, and microbiology. Annu Rev Pathol.

[50] Pang Y, Tan Y, Chen J, Li Y, Zheng H, Song Y, Zhao Y (2017). Diversity of nontuberculous mycobacteria in eastern and southern China: a cross-sectional study. Eur Respir J.

[51] Huang JJ, Li YX, Zhao Y, Yang WH, Xiao M, Kudinha T, Xu YC (2020). Prevalence of nontuberculous mycobacteria in a tertiary hospital in Beijing, China, January 2013 to December 2018. BMC Microbiol.

[52] Hoefsloot W, van Ingen J, Andrejak C, Angeby K, Bauriaud R, Bemer P, Beylis N, Boeree MJ, Cacho J, Chihota V (2013). The geographic diversity of nontuberculous mycobacteria isolated from pulmonary samples: an NTM-NET collaborative study. Eur Respir J.

[53] Chu H, Zhao L, Xiao H, Zhang Z, Zhang J, Gui T, Gong S, Xu L, Sun X (2014). Prevalence of nontuberculous mycobacteria in patients with bronchiectasis: a meta-analysis. Arch Med Sci.

